# K_3_Gd(PO_4_)_2_

**DOI:** 10.1107/S160053681003103X

**Published:** 2010-08-11

**Authors:** Dan Zhao, Fei Fei Li

**Affiliations:** a Department of Physics and Chemistry, Henan Polytechnic University, Jiaozuo, Henan 454000, People’s Republic of China

## Abstract

The title compound, tripotassium gadolinium(III) bis­[ortho­phosphate(V)], was synthesized by a high-temperature solution reaction. Of the 12 atoms of the asymmetric unit (1 × Gd, 2 × P, 3 × K, 6 × O), all but two O atoms (which are in general positions) lie on mirror planes. The crystal structure features sheets of composition [Gd(PO_4_)_2_]^3−^ which extend parallel to (100) and are built up from isolated PO_4_ tetra­hedra and GdO_7_ monocapped prisms through corner- and edge-sharing. The K^+^ ions, which have coordination numbers of 10, 9 and 11, help to stack the anionic sheets along [100] into a three-dimensional structure.

## Related literature

For the structures, properties and applications of phosphates with general formula *M*_3_ *RE*(PO_4_)_2_ (*M* = alkali metal, *RE* = rare earth metal), see: K_3_Lu(PO_4_)_2_ (Efremov *et al.*, 1981 ▶); K_3_(La_0.99_Nd_0.01_)(PO_4_)_2_ (Hong & Chinn, 1976 ▶); Na_3_Ce(PO_4_)_2_ (Karpov *et al.*, 1980 ▶); K_3_Eu(PO_4_)_2_ (Morozov *et al.*, 2001 ▶); K_3_Sm(PO_4_)_2_ (Toumi *et al.*, 1999 ▶); K_3_Ce(PO_4_)_2_ (Zah-Letho *et al.*, 1988 ▶).

## Experimental

### 

#### Crystal data

K_3_Gd(PO_4_)_2_
*M* *_r_* = 464.49
Monoclinic, 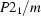

*a* = 7.4153 (15) Å
*b* = 5.6206 (11) Å
*c* = 9.445 (2) Å
β = 90.723 (14)°
*V* = 393.62 (14) Å^3^
*Z* = 2
Mo *K*α radiation
μ = 10.43 mm^−1^
*T* = 293 K
0.30 × 0.10 × 0.10 mm

#### Data collection

Rigaku Mercury70 CCD diffractometer
Absorption correction: multi-scan (*ABSCOR*; Higashi, 1995 ▶) *T*_min_ = 0.304, *T*_max_ = 0.624
3036 measured reflections
993 independent reflections
946 reflections with *I* > 2σ(*I*)
*R*_int_ = 0.045

#### Refinement

*R*[*F* ^2^ > 2σ(*F* ^2^)] = 0.027
*wR*(*F* ^2^) = 0.065
*S* = 1.03
993 reflections
80 parameters
Δρ_max_ = 2.38 e Å^−3^
Δρ_min_ = −2.68 e Å^−3^

### 

Data collection: *CrystalClear* (Rigaku, 2004 ▶); cell refinement: *CrystalClear*; data reduction: *CrystalClear*; program(s) used to solve structure: *SHELXS97* (Sheldrick, 2008 ▶); program(s) used to refine structure: *SHELXL97* (Sheldrick, 2008 ▶); molecular graphics: *DIAMOND* (Brandenburg, 2004 ▶); software used to prepare material for publication: *SHELXTL* (Sheldrick, 2008 ▶) and *PLATON* (Spek, 2009 ▶).

## Supplementary Material

Crystal structure: contains datablocks I, global. DOI: 10.1107/S160053681003103X/wm2378sup1.cif

Structure factors: contains datablocks I. DOI: 10.1107/S160053681003103X/wm2378Isup2.hkl

Additional supplementary materials:  crystallographic information; 3D view; checkCIF report

## Figures and Tables

**Fig. 1.**
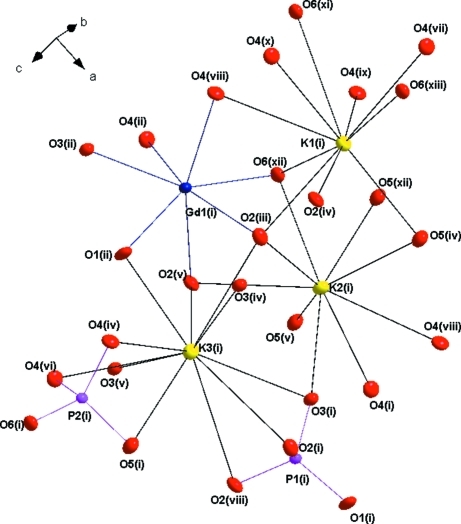
Section of the structure of K3Gd(PO4)2 with the atom labelling scheme. The displacement ellipsoids are drawn at the 50% probability level. [Symmetry codes: (i) x, y, z; (ii) -1+x, y, z; (iii) 1-x, 1-y, 1-z; (iv) 1-x, 0.5+y, 1-z; (v) 1-x, -0.5+y, 1-z; (vi) 1-x, -y, 1-z; (vii) 1-x, 1-y, -z; (viii) x, 0.5-y, z; (ix) 1-x, 0.5+y, -z; (x) -1+x, 1+y, z; (xi) -x, 0.5+y, 1-z; (xii) x, y, -1+z; (xiii) x, 1+y, -1+z.]

**Fig. 2.**
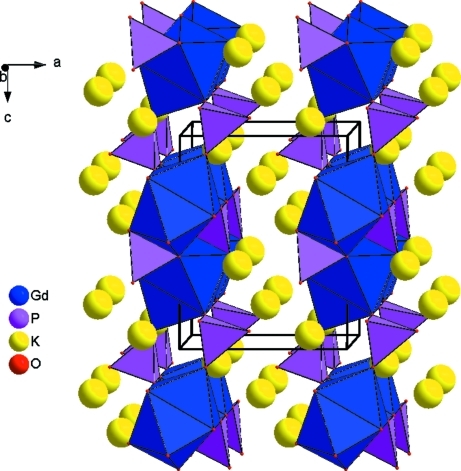
View of the crystal structure of K3Gd(PO4)2. K—O bonds were omitted for clarity.

## supplementary crystallographic information

### Comment

Inorganic phosphates with general formula *M*_3_*RE*(PO_4_)_2_ have been investigated in the past years due to their interesting optical properties, in which *M* is a alkali metal cation and *RE* is a trivalent rare-earth cation: K_3_Lu(PO_4_)_2_, space group *P*3 (Efremov *et al.*, 1981); K_3_(La_0.99_Nd_0.01_)(PO_4_)_2_, *P*2_1_/*m* (Hong & Chinn, 1976); Na_3_Ce(PO_4_)_2_, *Pca*2_1_ (Karpov *et al.*, 1980); K_3_Eu(PO_4_)_2_, *P*2_1_/*m* (Morozov *et al.*, 2001); K_3_Sm(PO_4_)_2_, *P*2_1_/*m* (Toumi *et al.*, 1999); K_3_Ce(PO_4_)_2_, *P*2_1_/*m* (Zah-Letho *et al.*, 1988). We report herein the synthesis and crystal structure of K_3_Gd(PO_4_)_2_ which is isotypic with all structures crystallizing in space group *P*2_1_/*m*.

As shown in Fig. 1, the crystal structure of K_3_Gd(PO_4_)_2_ features two-dimensional sheets with composition [Gd(PO_4_)_2_]^3-^ extending in the *bc* plane and constructed from isolated PO_4_ tetrahedra and isolated GdO_7_ monocapped prisms through corner- and edge-sharing. The K^+^ cations, with coordination numbers of 10 (K1), 9 (K2) and 11 (K3), are situated between these sheets and join them through coulombic action to the O^2-^ anions, eventually forming the three-dimensional framework of K_3_Gd(PO_4_)_2_ (Fig. 2).

### Experimental

The finely ground reagents K_2_CO_3_, Gd_2_O_3_, and NH_4_H_2_PO_4_ were mixed in the molar ratio K: Gd: P = 12: 1: 10, placed in a Pt crucible, and heated at 573 K for 4 h. The mixture was re-ground and heated at 1173 K for 20 h, then cooled to 573 K at a rate of 4 K h^-1^, and finally quenched to room temperature. A few colorless crystals of the title compound with prismatic shape were obtained.

### Refinement

The highest peak in the difference electron density map is 1.10 Å from Gd1 while the deepest hole is 0.85 Å from the same atom.

### Crystal data

**Table:**

K_3_Gd(PO_4_)_2_	*F*(000) = 430
*M**_r_* = 464.49	*D*_x_ = 3.919 Mg m^−^^3^
Monoclinic, *P*2_1_/*m*	Mo *K*α radiation, λ = 0.71073 Å
Hall symbol: -P 2yb	Cell parameters from 1264 reflections
*a* = 7.4153 (15) Å	θ = 2.2–27.5°
*b* = 5.6206 (11) Å	µ = 10.43 mm^−^^1^
*c* = 9.445 (2) Å	*T* = 293 K
β = 90.723 (14)°	Prism, colourless
*V* = 393.62 (14) Å^3^	0.30 × 0.10 × 0.10 mm
*Z* = 2	

### Data collection

**Table:**

Rigaku Mercury70 CCD diffractometer	993 independent reflections
Radiation source: fine-focus sealed tube	946 reflections with *I* > 2σ(*I*)
Graphite Monochromator	*R*_int_ = 0.045
Detector resolution: 14.6306 pixels mm^-1^	θ_max_ = 27.5°, θ_min_ = 2.2°
ω scans	*h* = −9→9
Absorption correction: multi-scan (*ABSCOR*; Higashi, 1995)	*k* = −7→7
*T*_min_ = 0.304, *T*_max_ = 0.624	*l* = −12→12
3036 measured reflections	

### Refinement

**Table:**

Refinement on *F*^2^	Primary atom site location: structure-invariant direct methods
Least-squares matrix: full	Secondary atom site location: difference Fourier map
*R*[*F*^2^ > 2σ(*F*^2^)] = 0.027	*w* = 1/[σ^2^(*F*_o_^2^) + (0.0356*P*)^2^] where *P* = (*F*_o_^2^ + 2*F*_c_^2^)/3
*wR*(*F*^2^) = 0.065	(Δ/σ)_max_ < 0.001
*S* = 1.03	Δρ_max_ = 2.38 e Å^−^^3^
993 reflections	Δρ_min_ = −2.68 e Å^−^^3^
80 parameters	Extinction correction: *SHELXL97* (Sheldrick, 2008), Fc^*^=kFc[1+0.001xFc^2^λ^3^/sin(2θ)]^-1/4^
0 restraints	Extinction coefficient: 0.0314 (17)

### Special details

**Table:**

Geometry. All e.s.d.'s (except the e.s.d. in the dihedral angle between two l.s. planes) are estimated using the full covariance matrix. The cell e.s.d.'s are taken into account individually in the estimation of e.s.d.'s in distances, angles and torsion angles; correlations between e.s.d.'s in cell parameters are only used when they are defined by crystal symmetry. An approximate (isotropic) treatment of cell e.s.d.'s is used for estimating e.s.d.'s involving l.s. planes.
Refinement. Refinement of *F*^2^ against ALL reflections. The weighted *R*-factor *wR* and goodness of fit *S* are based on *F*^2^, conventional *R*-factors *R* are based on *F*, with *F* set to zero for negative *F*^2^. The threshold expression of *F*^2^ > σ(*F*^2^) is used only for calculating *R*-factors(gt) *etc*. and is not relevant to the choice of reflections for refinement. *R*-factors based on *F*^2^ are statistically about twice as large as those based on *F*, and *R*- factors based on ALL data will be even larger.

### Fractional atomic coordinates and isotropic or equivalent isotropic displacement parameters (Å^2^)

**Table:**

	*x*	*y*	*z*	*U*_iso_*/*U*_eq_	
Gd1	0.00699 (3)	0.2500	0.29011 (3)	0.00731 (15)	
P1	0.80967 (19)	0.2500	0.57350 (15)	0.0076 (3)	
P2	0.2303 (2)	0.2500	0.91166 (15)	0.0066 (3)	
K1	0.20438 (17)	0.7500	0.08160 (13)	0.0119 (3)	
K2	0.50495 (17)	0.2500	0.19219 (13)	0.0142 (3)	
K3	0.36353 (18)	0.2500	0.59102 (14)	0.0142 (3)	
O1	1.0137 (6)	0.2500	0.5478 (4)	0.0169 (10)	
O2	0.7564 (4)	0.4735 (5)	0.6577 (3)	0.0123 (6)	
O3	0.7177 (6)	0.2500	0.4266 (4)	0.0116 (9)	
O4	0.8481 (4)	−0.0269 (5)	0.1623 (3)	0.0129 (6)	
O5	0.4337 (6)	0.2500	0.8991 (5)	0.0163 (9)	
O6	0.1744 (6)	0.2500	1.0689 (4)	0.0113 (9)	

### Atomic displacement parameters (Å^2^)

**Table:**

	*U*^11^	*U*^22^	*U*^33^	*U*^12^	*U*^13^	*U*^23^
Gd1	0.0066 (2)	0.0068 (2)	0.0085 (2)	0.000	−0.00097 (12)	0.000
P1	0.0065 (7)	0.0086 (8)	0.0076 (7)	0.000	−0.0012 (5)	0.000
P2	0.0070 (7)	0.0052 (7)	0.0076 (6)	0.000	−0.0009 (5)	0.000
K1	0.0137 (6)	0.0100 (7)	0.0118 (6)	0.000	−0.0001 (5)	0.000
K2	0.0101 (7)	0.0186 (8)	0.0138 (6)	0.000	−0.0021 (5)	0.000
K3	0.0126 (6)	0.0136 (7)	0.0165 (6)	0.000	−0.0002 (5)	0.000
O1	0.007 (2)	0.031 (3)	0.012 (2)	0.000	−0.0023 (16)	0.000
O2	0.0176 (16)	0.0087 (15)	0.0105 (14)	−0.0023 (12)	0.0007 (12)	−0.0019 (11)
O3	0.0087 (19)	0.015 (2)	0.0110 (19)	0.000	−0.0010 (16)	0.000
O4	0.0152 (15)	0.0081 (15)	0.0152 (14)	−0.0033 (12)	−0.0016 (12)	−0.0018 (12)
O5	0.010 (2)	0.021 (3)	0.018 (2)	0.000	0.0017 (16)	0.000
O6	0.013 (2)	0.014 (2)	0.0076 (19)	0.000	0.0012 (16)	0.000

### Geometric parameters (Å, °)

**Table:**

Gd1—O4^i^	2.287 (3)	K1—O4^xii^	3.030 (3)
Gd1—O4^ii^	2.287 (3)	K1—O6^xiii^	3.132 (4)
Gd1—O2^iii^	2.390 (3)	K2—O6^v^	2.700 (4)
Gd1—O2^iv^	2.390 (3)	K2—O3	2.702 (4)
Gd1—O1^i^	2.434 (4)	K2—O5^v^	2.812 (4)
Gd1—O6^v^	2.444 (4)	K2—O2^iv^	2.872 (3)
Gd1—O3^i^	2.517 (4)	K2—O2^iii^	2.872 (3)
P1—O1	1.535 (4)	K2—O5^vii^	2.9761 (15)
P1—O3	1.538 (4)	K2—O5^iii^	2.9761 (16)
P1—O2	1.541 (3)	K2—O4^vi^	2.999 (3)
P1—O2^vi^	1.541 (3)	K2—O4	2.999 (3)
P2—O5	1.515 (4)	K3—O1^i^	2.621 (4)
P2—O6	1.547 (4)	K3—O3^vii^	2.8785 (11)
P2—O4^vii^	1.545 (3)	K3—O3^iii^	2.8785 (11)
P2—O4^viii^	1.545 (3)	K3—O2^iv^	2.945 (3)
K1—O5^iii^	2.687 (5)	K3—O2^iii^	2.945 (3)
K1—O2^viii^	2.776 (3)	K3—O5	2.949 (4)
K1—O2^iii^	2.776 (3)	K3—O3	3.067 (4)
K1—O4^ix^	2.803 (3)	K3—O4^viii^	3.092 (3)
K1—O4^x^	2.803 (3)	K3—O4^vii^	3.092 (3)
K1—O6^v^	2.8215 (7)	K3—O2	3.227 (3)
K1—O6^xi^	2.8215 (7)	K3—O2^vi^	3.227 (3)
K1—O4^ii^	3.030 (3)		
			
O4^i^—Gd1—O4^ii^	85.75 (15)	O3^vii^—K3—O5	95.24 (9)
O4^i^—Gd1—O2^iii^	157.36 (10)	O3^iii^—K3—O5	95.24 (9)
O4^ii^—Gd1—O2^iii^	92.20 (11)	O2^iv^—K3—O5	146.60 (6)
O4^i^—Gd1—O2^iv^	92.20 (11)	O2^iii^—K3—O5	146.60 (6)
O4^ii^—Gd1—O2^iv^	157.36 (10)	O1^i^—K3—O3	140.64 (13)
O2^iii^—Gd1—O2^iv^	81.09 (14)	O3^vii^—K3—O3	98.65 (8)
O4^i^—Gd1—O1^i^	122.12 (9)	O3^iii^—K3—O3	98.65 (8)
O4^ii^—Gd1—O1^i^	122.12 (9)	O2^iv^—K3—O3	81.26 (9)
O2^iii^—Gd1—O1^i^	77.78 (10)	O2^iii^—K3—O3	81.26 (9)
O2^iv^—Gd1—O1^i^	77.78 (10)	O5—K3—O3	110.96 (12)
O4^i^—Gd1—O6^v^	79.20 (10)	O1^i^—K3—O4^viii^	66.82 (11)
O4^ii^—Gd1—O6^v^	79.20 (10)	O3^vii^—K3—O4^viii^	109.38 (10)
O2^iii^—Gd1—O6^v^	78.27 (10)	O3^iii^—K3—O4^viii^	62.60 (10)
O2^iv^—Gd1—O6^v^	78.27 (10)	O2^iv^—K3—O4^viii^	131.63 (9)
O1^i^—Gd1—O6^v^	148.30 (14)	O2^iii^—K3—O4^viii^	103.66 (8)
O4^i^—Gd1—O3^i^	80.43 (10)	O5—K3—O4^viii^	48.81 (9)
O4^ii^—Gd1—O3^i^	80.43 (10)	O3—K3—O4^viii^	145.84 (9)
O2^iii^—Gd1—O3^i^	121.51 (9)	O1^i^—K3—O4^vii^	66.82 (11)
O2^iv^—Gd1—O3^i^	121.51 (9)	O3^vii^—K3—O4^vii^	62.60 (10)
O1^i^—Gd1—O3^i^	59.63 (13)	O3^iii^—K3—O4^vii^	109.38 (10)
O6^v^—Gd1—O3^i^	152.07 (14)	O2^iv^—K3—O4^vii^	103.66 (8)
O1—P1—O3	106.5 (2)	O2^iii^—K3—O4^vii^	131.63 (9)
O1—P1—O2	109.94 (15)	O5—K3—O4^vii^	48.81 (9)
O3—P1—O2	110.60 (15)	O3—K3—O4^vii^	145.84 (9)
O1—P1—O2^vi^	109.94 (15)	O4^viii^—K3—O4^vii^	47.86 (11)
O3—P1—O2^vi^	110.60 (15)	O1^i^—K3—O2	156.87 (6)
O2—P1—O2^vi^	109.2 (2)	O3^vii^—K3—O2	125.38 (10)
O5—P2—O6	110.7 (2)	O3^iii^—K3—O2	79.56 (10)
O5—P2—O4^vii^	109.50 (16)	O2^iv^—K3—O2	128.60 (6)
O6—P2—O4^vii^	109.28 (15)	O2^iii^—K3—O2	102.30 (8)
O5—P2—O4^viii^	109.50 (16)	O5—K3—O2	70.11 (10)
O6—P2—O4^viii^	109.28 (15)	O3—K3—O2	47.34 (8)
O4^vii^—P2—O4^viii^	108.5 (2)	O4^viii^—K3—O2	99.24 (8)
O5^iii^—K1—O2^viii^	81.16 (11)	O4^vii^—K3—O2	118.45 (9)
O5^iii^—K1—O2^iii^	81.16 (11)	P2—K3—O2	95.98 (7)
O2^viii^—K1—O2^iii^	53.82 (12)	O1^i^—K3—O2^vi^	156.87 (6)
O5^iii^—K1—O4^ix^	100.61 (10)	O3^vii^—K3—O2^vi^	79.56 (10)
O2^viii^—K1—O4^ix^	172.75 (9)	O3^iii^—K3—O2^vi^	125.38 (10)
O2^iii^—K1—O4^ix^	119.30 (8)	O2^iv^—K3—O2^vi^	102.30 (8)
O5^iii^—K1—O4^x^	100.61 (10)	O2^iii^—K3—O2^vi^	128.60 (6)
O2^viii^—K1—O4^x^	119.30 (8)	O5—K3—O2^vi^	70.11 (10)
O2^iii^—K1—O4^x^	172.75 (9)	O3—K3—O2^vi^	47.34 (8)
O4^ix^—K1—O4^x^	67.45 (12)	O4^viii^—K3—O2^vi^	118.45 (9)
O5^iii^—K1—O6^v^	94.64 (9)	O4^vii^—K3—O2^vi^	99.24 (8)
O2^viii^—K1—O6^v^	119.72 (11)	P2—K3—O2^vi^	95.98 (7)
O2^iii^—K1—O6^v^	66.06 (10)	O2—K3—O2^vi^	45.82 (11)
O4^ix^—K1—O6^v^	53.27 (10)	P1—O1—Gd1^xiv^	98.6 (2)
O4^x^—K1—O6^v^	120.53 (11)	P1—O1—K3^xiv^	162.0 (3)
O5^iii^—K1—O6^xi^	94.64 (9)	Gd1^xiv^—O1—K3^xiv^	99.39 (14)
O2^viii^—K1—O6^xi^	66.06 (10)	P1—O2—Gd1^iii^	116.36 (16)
O2^iii^—K1—O6^xi^	119.72 (11)	P1—O2—K1^iii^	93.68 (14)
O4^ix^—K1—O6^xi^	120.53 (11)	Gd1^iii^—O2—K1^iii^	92.46 (10)
O4^x^—K1—O6^xi^	53.27 (10)	P1—O2—K2^iii^	151.02 (17)
O6^v^—K1—O6^xi^	169.79 (18)	Gd1^iii^—O2—K2^iii^	92.56 (9)
O5^iii^—K1—O4^ii^	148.85 (8)	K1^iii^—O2—K2^iii^	82.58 (8)
O2^viii^—K1—O4^ii^	92.65 (9)	P1—O2—K3^iii^	95.46 (13)
O2^iii^—K1—O4^ii^	70.83 (9)	Gd1^iii^—O2—K3^iii^	92.00 (9)
O4^ix^—K1—O4^ii^	82.21 (9)	K1^iii^—O2—K3^iii^	166.79 (12)
O4^x^—K1—O4^ii^	108.90 (7)	K2^iii^—O2—K3^iii^	84.80 (9)
O6^v^—K1—O4^ii^	61.97 (10)	P1—O2—K3	79.54 (13)
O6^xi^—K1—O4^ii^	110.76 (11)	Gd1^iii^—O2—K3	162.12 (12)
O5^iii^—K1—O4^xii^	148.85 (8)	K1^iii^—O2—K3	94.66 (9)
O2^viii^—K1—O4^xii^	70.83 (9)	K2^iii^—O2—K3	72.18 (7)
O2^iii^—K1—O4^xii^	92.65 (9)	K3^iii^—O2—K3	77.70 (8)
O4^ix^—K1—O4^xii^	108.90 (7)	P1—O3—Gd1^xiv^	95.21 (19)
O4^x^—K1—O4^xii^	82.21 (9)	P1—O3—K2	170.6 (2)
O6^v^—K1—O4^xii^	110.76 (11)	Gd1^xiv^—O3—K2	94.17 (13)
O6^xi^—K1—O4^xii^	61.97 (10)	P1—O3—K3^vii^	98.18 (9)
O4^ii^—K1—O4^xii^	48.89 (11)	Gd1^xiv^—O3—K3^vii^	98.59 (8)
O5^iii^—K1—O6^xiii^	156.91 (13)	K2—O3—K3^vii^	80.39 (9)
O2^viii^—K1—O6^xiii^	119.07 (9)	P1—O3—K3^iii^	98.18 (9)
O2^iii^—K1—O6^xiii^	119.07 (9)	Gd1^xiv^—O3—K3^iii^	98.59 (8)
O4^ix^—K1—O6^xiii^	60.83 (9)	K2—O3—K3^iii^	80.39 (9)
O4^x^—K1—O6^xiii^	60.83 (9)	K3^vii^—O3—K3^iii^	155.01 (17)
O6^v^—K1—O6^xiii^	84.90 (9)	P1—O3—K3	85.20 (16)
O6^xi^—K1—O6^xiii^	84.90 (9)	Gd1^xiv^—O3—K3	179.59 (17)
O4^ii^—K1—O6^xiii^	48.27 (9)	K2—O3—K3	85.42 (12)
O4^xii^—K1—O6^xiii^	48.27 (9)	K3^vii^—O3—K3	81.34 (8)
O6^v^—K2—O3	150.53 (13)	K3^iii^—O3—K3	81.34 (8)
O6^v^—K2—O5^v^	54.35 (12)	P2^vii^—O4—Gd1^xiv^	168.2 (2)
O3—K2—O5^v^	155.12 (13)	P2^vii^—O4—K1^x^	91.81 (14)
O6^v^—K2—O2^iv^	66.32 (9)	Gd1^xiv^—O4—K1^x^	96.97 (10)
O3—K2—O2^iv^	89.20 (10)	P2^vii^—O4—K2	98.57 (15)
O5^v^—K2—O2^iv^	111.49 (10)	Gd1^xiv^—O4—K2	91.67 (10)
O6^v^—K2—O2^iii^	66.32 (9)	K1^x^—O4—K2	71.34 (8)
O3—K2—O2^iii^	89.20 (10)	P2^vii^—O4—K1^xv^	82.80 (13)
O5^v^—K2—O2^iii^	111.49 (10)	Gd1^xiv^—O4—K1^xv^	88.25 (10)
O2^iv^—K2—O2^iii^	65.51 (12)	K1^x^—O4—K1^xv^	97.79 (9)
O6^v^—K2—O5^vii^	90.93 (9)	K2—O4—K1^xv^	169.05 (11)
O3—K2—O5^vii^	98.48 (9)	P2^vii^—O4—K3^vii^	79.62 (13)
O5^v^—K2—O5^vii^	75.07 (9)	Gd1^xiv^—O4—K3^vii^	98.11 (10)
O2^iv^—K2—O5^vii^	74.84 (10)	K1^x^—O4—K3^vii^	141.08 (12)
O2^iii^—K2—O5^vii^	139.49 (10)	K2—O4—K3^vii^	72.55 (7)
O6^v^—K2—O5^iii^	90.93 (9)	K1^xv^—O4—K3^vii^	118.30 (10)
O3—K2—O5^iii^	98.48 (9)	P2—O5—K1^iii^	171.6 (3)
O5^v^—K2—O5^iii^	75.07 (9)	P2—O5—K2^xvi^	95.6 (2)
O2^iv^—K2—O5^iii^	139.49 (10)	K1^iii^—O5—K2^xvi^	76.00 (12)
O2^iii^—K2—O5^iii^	74.84 (10)	P2—O5—K3	85.06 (19)
O5^vii^—K2—O5^iii^	141.57 (16)	K1^iii^—O5—K3	103.34 (14)
O6^v^—K2—O4^vi^	136.55 (9)	K2^xvi^—O5—K3	179.34 (17)
O3—K2—O4^vi^	65.80 (10)	P2—O5—K2^vii^	100.29 (10)
O5^v^—K2—O4^vi^	93.23 (10)	K1^iii^—O5—K2^vii^	82.16 (9)
O2^iv^—K2—O4^vi^	154.93 (10)	K2^xvi^—O5—K2^vii^	104.93 (9)
O2^iii^—K2—O4^vi^	110.14 (8)	K3—O5—K2^vii^	74.93 (9)
O5^vii^—K2—O4^vi^	109.26 (11)	P2—O5—K2^iii^	100.29 (10)
O5^iii^—K2—O4^vi^	49.45 (10)	K1^iii^—O5—K2^iii^	82.16 (9)
O6^v^—K2—O4	136.55 (9)	K2^xvi^—O5—K2^iii^	104.93 (9)
O3—K2—O4	65.80 (9)	K3—O5—K2^iii^	74.93 (9)
O5^v^—K2—O4	93.23 (10)	K2^vii^—O5—K2^iii^	141.57 (16)
O2^iv^—K2—O4	110.14 (8)	P2—O6—Gd1^xvi^	165.0 (3)
O2^iii^—K2—O4	154.93 (10)	P2—O6—K2^xvi^	99.3 (2)
O5^vii^—K2—O4	49.45 (10)	Gd1^xvi^—O6—K2^xvi^	95.71 (14)
O5^iii^—K2—O4	109.26 (11)	P2—O6—K1^xvi^	91.08 (8)
O4^vi^—K2—O4	62.52 (12)	Gd1^xvi^—O6—K1^xvi^	90.24 (8)
O1^i^—K3—O3^vii^	77.59 (8)	K2^xvi^—O6—K1^xvi^	84.90 (9)
O1^i^—K3—O3^iii^	77.59 (8)	P2—O6—K1^xvii^	91.08 (8)
O3^vii^—K3—O3^iii^	155.01 (17)	Gd1^xvi^—O6—K1^xvii^	90.24 (8)
O1^i^—K3—O2^iv^	65.64 (10)	K2^xvi^—O6—K1^xvii^	84.90 (9)
O3^vii^—K3—O2^iv^	51.52 (10)	K1^xvi^—O6—K1^xvii^	169.79 (18)
O3^iii^—K3—O2^iv^	114.09 (11)	P2—O6—K1^xiii^	79.26 (17)
O1^i^—K3—O2^iii^	65.64 (10)	Gd1^xvi^—O6—K1^xiii^	85.73 (12)
O3^vii^—K3—O2^iii^	114.09 (11)	K2^xvi^—O6—K1^xiii^	178.56 (15)
O3^iii^—K3—O2^iii^	51.52 (10)	K1^xvi^—O6—K1^xiii^	95.10 (9)
O2^iv^—K3—O2^iii^	63.70 (12)	K1^xvii^—O6—K1^xiii^	95.10 (9)
O1^i^—K3—O5	108.39 (13)		

Symmetry codes: (i) *x*−1, *y*, *z*; (ii) *x*−1, −*y*+1/2, *z*; (iii) −*x*+1, −*y*+1, −*z*+1; (iv) −*x*+1, *y*−1/2, −*z*+1; (v) *x*, *y*, *z*−1; (vi) *x*, −*y*+1/2, *z*; (vii) −*x*+1, −*y*, −*z*+1; (viii) −*x*+1, *y*+1/2, −*z*+1; (ix) −*x*+1, *y*+1/2, −*z*; (x) −*x*+1, −*y*+1, −*z*; (xi) *x*, *y*+1, *z*−1; (xii) *x*−1, *y*+1, *z*; (xiii) −*x*, −*y*+1, −*z*+1; (xiv) *x*+1, *y*, *z*; (xv) *x*+1, *y*−1, *z*; (xvi) *x*, *y*, *z*+1; (xvii) *x*, *y*−1, *z*+1.
